# Phytochemical Profile and Evaluation of the Antioxidant, Cyto-Genotoxic, and Antigenotoxic Potential of *Salvia verticillata* Hydromethanolic Extract

**DOI:** 10.3390/plants13050731

**Published:** 2024-03-05

**Authors:** Lamprini S. Stavropoulou, Ioanna Efthimiou, Lambrini Giova, Chrysoula Manoli, Paraskevi S. Sinou, Aris Zografidis, Fotini N. Lamari, Dimitris Vlastos, Stefanos Dailianis, Maria Antonopoulou

**Affiliations:** 1 Laboratory of Pharmacognosy & Chemistry of Natural Products, Department of Pharmacy, University of Patras, GR-26504 Patras, Greece; stavropoulou.lamprini@gmail.com (L.S.S.); paraskevi.sinou@gmail.com (P.S.S.); flam@upatras.gr (F.N.L.); 2 Department of Biology, School of Natural Sciences, University of Patras, GR-26504 Patras, Greece; iefthimiou@upatras.gr (I.E.); lgiova@ac.upatras.gr (L.G.); up1073899@upnet.gr (C.M.); dvlastos@upatras.gr (D.V.); sdailianis@upatras.gr (S.D.); 3 Laboratory of Botany, Department of Biology, University of Patras, GR-26504 Patras, Greece; azografidis@upatras.gr; 4 Department of Sustainable Agriculture, University of Patras, GR-30131 Agrinio, Greece

**Keywords:** *Salvia verticillata*, rosmarinic acid, antioxidant activity, CBMN assay, cytotoxicity, antigenotoxicity, human lymphocytes

## Abstract

This study comprises the phytochemical characterization, the evaluation of the total phenolic content (TPC) and antioxidant activity (AA), and the investigation of the cyto-genotoxic and antigenotoxic potential of hydromethanolic extract derived from *Salvia verticillata* L. leaves. HPLC–DAD–ESI-MS and HPLC–DAD were used for the characterization of the extract and determination of the major ingredients. Afterwards, the TPC and AA were determined. The cytotoxic and genotoxic effect of the extract on cultured human lymphocytes at concentrations of 10, 25, and 50 μg mL^−1^ was investigated via the Cytokinesis Block MicroNucleus (CBMN) assay. Moreover, its antigenotoxic potential against the mutagenic agent mitomycin C (MMC) was assessed using the same assay. The hydromethanolic extract comprises numerous metabolites, with rosmarinic acid being the major compound. It had a high value of TPC and exerted significant AA as shown by the results of the Ferric Reducing Antioxidant Power (FRAP) and Radical Scavenging Activity by DPPH• assays. A dose-dependent cytotoxic potential was recorded, with the highest dose (50 μg mL^−1^) exhibiting statistically significant cytotoxicity. None of the tested concentrations induced significant micronuclei (MN) frequencies, indicating a lack of genotoxicity. All tested concentrations reduced the MMC-mediated genotoxic effects, with the two lowest showing statistically significant antigenotoxic potential.

## 1. Introduction

The genus *Salvia*, belonging to the *Lamiaceae* family, comprises more than a thousand species and has worldwide distribution, being mainly found in Central and East Asia, Central and South America, and the Mediterranean regions [1,2]. *Salvia verticillata* L., also known as lilac sage or whorled clary, is mostly distributed in Europe and Asia [3]. Some *Salvia* species, including *S. verticillata,* have been used in local cuisines, as ornamental plants, and in folk medicine [4], mostly for their antiseptic and anti-inflammatory properties [5]. Sage plants are aromatic, and their phytochemistry is rich since they contain several phenolic acid derivatives, flavonoids, and terpenes in high amounts, which contribute to their beneficial antimicrobial, antioxidant, anti-dementia, antiproliferative, and anticancer activities, amongst others [4,6]. Compared to *Salvia officinalis* L., or even to other more restricted species like *Salvia fruticosa* Mill., *S. verticillata* has not been largely studied, although there is a soaring demand for sage raw material and final products in the food, perfume, pharmaceutical, and cosmetic industries [4]. Most previous studies have studied the essential oils of wild *S. verticillata*, while those focusing on the analysis of polar metabolites of various parts of *S. verticillata* have shown that it contains numerous phenols (with rosmarinic acid predominant), flavonoids, and terpenoids [7,8]. Needless to say, sage phytochemical composition largely changes according to the genotype, the environmental conditions, the time of collection, and the plant part used; sage leaves constitute the herbal drug and raw material for most applications [9,10]. No study so far has reported a phytochemical analysis of the leaf extract of wild *S. verticillata* from Greece.

Efforts to cultivate *Salvia verticillata* as an alternative sage species are showing up [3,11,12]. Some studies have demonstrated the antioxidant, anti-inflammatory, antimicrobial, anti-acetylcholinesterase, anti-glucosidase, antityrosinase, cytotoxic, anticonvulsant, antidepressant, and nootropic properties of the *S. verticillata* aerial parts extract [1,7,8,13], while its aqueous extract has been used for the production of silver nanoparticles with antibacterial and cytotoxic activities [14,15,16,17,18]. Considering the increasing research interest in *S. verticillata* extracts and their potential use in beverages, as food ingredients/preservatives, cosmetic ingredients, or herbal supplements, it is of great importance to ensure their safety. To this end, this study focused on a thorough phytochemical analysis of the hydromethanolic extract of *S. verticillata* leaves native to the Peloponnese area in Greece, the investigation of its antioxidant potential, and the assessment of its cyto-genotoxic and antigenotoxic activity on human lymphocytes. Specifically, HPLC–DAD–ESI-MS was used for the analysis of the extract and the quantification process was conducted with HPLC–DAD. Afterward, the total phenolic content (TPC) of the extract was determined, followed by the investigation of its antioxidant activity via the FRAP and DPPH• scavenging assays. Subsequently, the Cytokinesis Block MicroNucleus (CBMN) assay was applied to peripheral blood lymphocytes to assess the cyto-genotoxic potential of the extract (10, 25, and 50 μg mL^−1^), as well as its antigenotoxic potential against the mutagenic agent mitomycin C (MMC). In fact, the CBMN assay is a rapid, sensitive, and reliable test, which can be used to detect micronuclei (MN) in the cytoplasm of interphase cells [19]. MN formation results from the inability of acentric fragments or whole chromosomes to migrate with the rest of the chromosomes during the anaphase of cell division, and the frequency of MN is an indicator of the genotoxic activity of the tested compound. Furthermore, the cytotoxic potential of said compound can also be assessed through the determination of the cytokinesis-block proliferation index (CBPI) [20,21].

## 2. Results and Discussion

### 2.1. Determination of Phenolic, Flavone, and Diterpene Metabolites in Hydromethanolic Extracts of S. verticillata

The yield of hydromethanolic extracts of *Salvia verticillata* was 16.41 ± 2.54% dry extract weight per 100 g of dry leaf weight (*n* = 3). The extracts were analyzed through HPLC–DAD–ESI-MS using both positive and negative ionization modes. Representative chromatograms are presented in Supplementary Figure S1. The characterization of the secondary metabolites, based on their MS and UV–vis spectra, their elution order on a reversed-phase C18 column, and the relevant literature ([7,8,22,23,24,25,26,27,28,29,30,31,32,33,34,35]), are presented in Table 1, while the elution times and the spectral characteristics of the unknown compounds are presented in Table S1.

Caffeic acid is involved in the biosynthesis of secondary metabolites in the Lamiaceae family and in sage plants in particular, and it is found primarily in its ester with 3,4-dihydroxy phenyl lactic acid (rosmarinic acid). Though the main representative group of the genus *Salvia* is that of phenolic acids, other classes of the polyphenolic spectrum such as flavones, flavonols, and their glycosides are present in *S. verticillata* extract [7].

Twenty-eight (28) compounds were detected in the *S. verticillata* extract, with the majority (thirteen) being phenolic acids (mainly caffeic acid derivatives) like caftaric acid, dimer-β-(3,4-dihydroxyphenyl) lactic acid, sagerinic acid, methyl-rosmarinic acid, salvianolic acids, and rosmarinic acid, which was the main ingredient in other *Salvia* species as previously demonstrated by our research group [22]. Moreover, flavones (five) were also detected in substantial amounts, such as glucuronides of luteolin, apigenin and hispidulin, circimaritin, and salvigenin. One organic acid (peak 1), one lignan (medioresinol), and one diterpene (peak 28) were also identified. The extract was rich in unidentified compounds (seven) believed to be mainly caffeic acid derivatives according to their UV–vis absorption spectra (Table S1), while the unknown compound 8 (MW = 592) was one of the main components of the extract and thus quantifiable (13.50 ± 1.63 mg g^−1^ dry extract).

Altogether, the common ingredients between our study and that of Katanić Stanković [7] are coumaroyl hexose, rosmarinic acid and its methyl ester, luteolin and apigenin hexuronides, and cirsimaritin. Possible differences could be due to the extraction process (leaves rather than aerial parts were extracted with 70% methanol), the different geographical origin (South Greece vs. Serbia), and therefore the different environmental conditions. Similarly, despite differences in plant material (leaves vs. aerial parts), extraction solvent (70% methanol vs. methanol), and geographical origin (Greece vs. Turkey), coumaroyl hexose, luteolin glucuronide, sagerinic acid, rosmarinic acid and its methyl esters, and salvianolic acid K were common in our study and in that of Zengin et al. [8]

In total, in this study, four phenolic acid derivatives and five flavonoids were quantified (Table 2, Figure S2).

Rosmarinic acid, being the main component, was quantified at 223.12 ± 8.66 mg g^−1^ dry extract weight, similar to the literature for *S. verticillata* extracts [7,8,36]. Compared to our previous findings on *Salvia* species, rosmarinic acid content was remarkably high, reinforcing earlier suggestions that *S. verticillata* is one of the species with the highest concentrations [8]. The latter could also explain the extract’s high concentration of *trans*-methyl rosmarinic acid at 63.03 ± 7.33 mg g^−1^ dry extract, which was not present in our previous study on different *Salvia* species and is substantially higher than that previously reported for *S. verticillata* [7,22]. The extract also had an important amount of dedihydro-salvianolic acid B/isomer, which has only been reported in *Salvia* species once [33].

Abietane diterpenes, such as carnosol and carnosic acid, were not detected, unlike in other *Salvia* species investigated in the past [22,28]. In the study by Katanić Stanković et al. [7], those were quantified in the *S. verticillata* extract, albeit in a very small quantity compared to the other acids, and thus we may not detect them because of lack of sufficient sensitivity. To our knowledge, this study is the first investigation of the polar metabolites of a population of *Salvia verticillata* L. from the Peloponnese in Greece, and it is in great accordance with previous findings. The differences noted with other studies denote the significance of the phytochemical screening of different genotypes from different geographical areas.

### 2.2. Total Phenolic Content and Antioxidant Activity

The hydromethanolic extract of *Salvia verticillata* was investigated for its total phenolic content and antioxidant activity using in vitro assays. According to our findings, the scavenging potential of the extract towards the DPPH free radical (IC_50_ = 15.8 μg/mL) was higher than in other studies on *S. verticillata* polar extracts in the past [7,36,37], higher than that of butylated hydroxytoluene (BHT), which was used as a reference standard (IC_50_ = 94.6 μg/mL) in this study, and higher than in other *Salvia* species like *S. officinalis*, *Salvia sclarea* L., and *Salvia euphratica* Montbret and Aucher [13,38]. Moreover, the extract is rich in phenolics, the content of which (359.6 ± 11.7 mg gallic acid equivalents/g dry extract) is higher than in other studies of *S. verticillata* polar extracts and other *Salvia* taxa extracts as well [8,39]. According to Kılıçkaya Selvi et al. [39], *Salvia tomentosa* Mill. possesses three times less phenolic content than *S. verticillata*. The Ferric Reducing Antioxidant Power (FRAP) assay follows the same pattern as the DPPH assay, and our results are 1192.1 ± 91.6 mg FeSO_4_ H_2_O/g DE.

### 2.3. CBMN Assay in Human Lymphocytes

**Figure 1:**
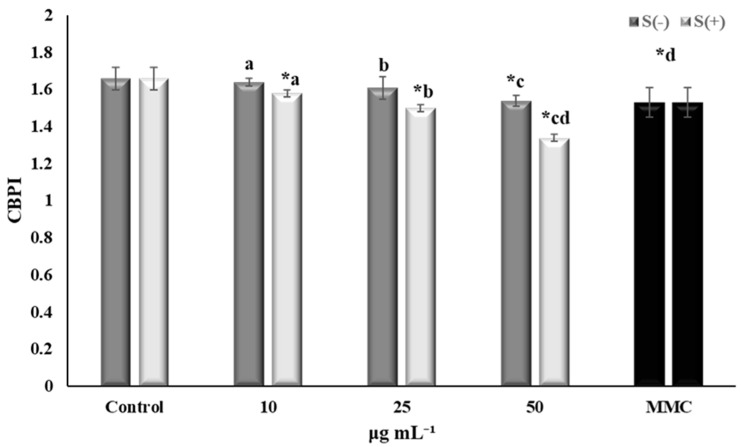
Cytotoxicity (in terms of CBPI values) of *Salvia verticillata* (S) extracts in human lymphocytes with (+) or without (−) mitomycin C (MMC, 0.05 μg mL^−1^). Samples sharing the same letter have a significant difference from each other. Asterisks (*) reveal a significant difference from control (*p* < 0.05).

**Figure 2:**
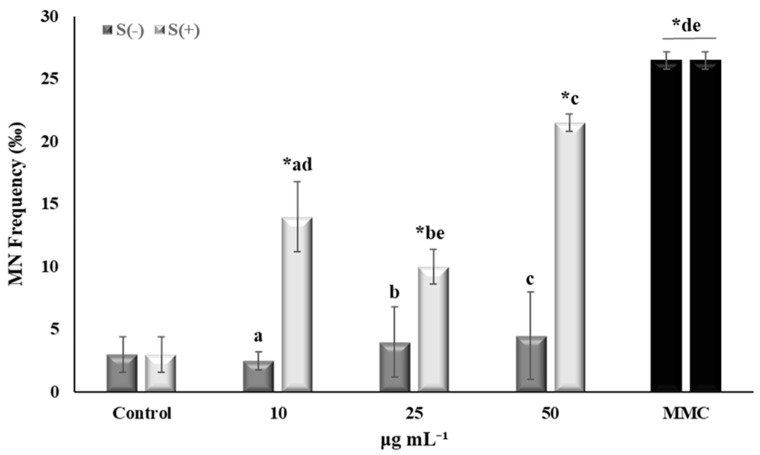
Micronuclei formation in human lymphocytes treated with *Salvia verticillata* (S) extracts with (+) or without (−) mitomycin C (MMC, 0.05 μg mL^−1^). Samples sharing the same letter have a significant difference from each other. Asterisks (*) reveal a significant difference from control (*p* < 0.05).

The *Salvia verticillata* extract was tested for its cytotoxic and genotoxic potential at three different concentrations (10, 25, and 50 μg mL^−1^). Moreover, its ability to decrease MMC-mediated cytotoxic and genotoxic effects on human lymphocytes was assessed (Figure 1 and Figure 2).

#### 2.3.1. Cytotoxic Activity

The cytotoxic potential of the extract (in terms of CBPI values) was investigated both in the presence and absence of MMC (Figure 1). According to the results, a dose-dependent decrease of the CBPI was recorded, with the highest concentration of *S. verticillata* extract (50 μg mL^−1^) exhibiting statistically significant cytotoxicity compared to the negative control. MMC-treated cells, with or without the extract, exhibited cytotoxic activity in all concentrations tested. Plants and their metabolites constitute valuable candidates for the development of anticancer agents. It has been stated that more than 60% of anticancer drugs are derived from natural products [40]. Similarly, *S. verticillata* has been investigated for its cytotoxic and anticancer potential in previous studies. Methanol and water extracts of the aerial parts of *S. verticillata* subsp. *amasica* were evaluated via the 3-(4,5-dimethylthiazol-2-yl)-2,5-diphenyl tetrazolium bromide (MTT) colorimetric assay for their cytotoxic potential against two cell lines (human alveolar lung epithelial carcinoma (A549) and human breast adenocarcinoma (MCF-7)). Moderate time- and dose-dependent cytotoxic activity was observed, which was attributed to phytochemical composition. Specifically, the IC_50_ values of the water extract were 460 ± 11 (24 h), 289 ± 9.6 (48 h), and 156 ± 4.1 (72 h) μg/mL against A549 cells, and 500 ± 14.6 (24 h), 279 ± 7.7 (48 h), and 224 ± 5.6 (72 h) μg/mL against MCF-7 cells. The methanol extract was less cytotoxic against A549 cells with IC_50_ values of 835 ± 24 (24 h), 540 ± 19 (48 h), and 302 ± 9.2 (72 h) μg/mL. IC_50_ values against MCF-7 cells were 325 ± 5.9 (24 h), 407 ± 13 (48 h), and 312 ± 7.8 (72 h) μg/mL. Significant antitumor activity (88% inhibition) was exhibited by the methanolic extract of *S. verticillata* via the *Agrobacterium tumefaciens*-induced potato disk tumor assay [41]. On the other hand, methanolic extract from *S. verticillata* aerial parts was investigated for its cytotoxicity against Murine BALBC-3T3 (fibroblasts), human A431 (epidermoid carcinoma), HepG2 (hepatic carcinoma), and LoVo (colorectal adenocarcinoma) cells, using the MTT assay. According to the results, the cell viability was not affected in any case at 48 h, followed by a slight decrease over time (72 h), at least in the case of LoVo cancer cells at 50 μg/mL [7]. However, it is well known that the use of different assays, cell lines, and experimental conditions can lead to different results. The cytotoxic potential of natural products has been linked to their compounds, including phenols and flavonoids [42]. Given that rosmarinic acid is the main component of the hydromethanolic extract, its contribution to the obtained cytotoxicity should not be ruled out. In fact, rosmarinic acid has been reported to exhibit cytotoxic effects on human Hep-G2 liver carcinoma cells [43], ARH-77 human (multiple myeloma) cells [44], and prostate cancer cells [45]; antitumor effects on 7,12-dimethylbenz(a)anthracene-induced skin carcinogenesis [46]; and inhibitory effects on the metastatic properties of colorectal cancer cells [47]. Thus, the hydromethanolic extract derived from *Salvia verticillata* in our study exhibited cytotoxic potential, in accordance with previous research on different *Salvia verticillata* extracts.

#### 2.3.2. Genotoxic and Antigenotoxic Activity

Regarding the extracts’ genotoxic potential, low MN frequencies were recorded in cells treated with all concentrations tested (10, 25, and 50 μg mL^−1^) compared to control, thus indicating the absence of genotoxicity (Figure 2). Although different species from the *Salvia* genus have been examined for their genotoxic activity, this study is the first to show the absence of a genotoxic potential in our hydromethanolic *S. verticillata* extract. Similarly, ethanol extract from the aerial parts of *S. hypargeia* at 0.5 and 1% *w*/*w* did not lead to increased MN frequencies on rat peripheral blood samples, according to Ozay et al. [48]. On the other hand, MMC-treated cells exhibited increased MN frequencies as expected, confirming genotoxic action. MMC is an antitumor, antibiotic agent which has been proven to exert several genotoxic effects including mutagenesis, clastogenesis, and inhibition of DNA synthesis. Its status as a carcinogenic agent has been demonstrated in all in vitro and in vivo test systems in mammalian cells and animals [49]. Interestingly, mixtures of MMC with 10 and 25 μg mL^−1^ of the extract led to the attenuation of MMC-mediated genotoxic effects. These findings are in accordance with Oalđe Pavlović et al. [50], who showed the significant antigenotoxic ability of the methanolic extract of *S. officinalis* against hydrogen peroxide in the human fetal lung fibroblast (MRC-5) cell line at the lowest concentration tested, i.e., 25 μg/mL. Moreover, there is evidence that *S. officinalis* extract from leaves, in the form of a tea infusion, exerts antimutagenic activity against the mutagen methyl methanesulphonate, using the somatic mutation and recombination test (SMART) on *Drosophila melanogaster* [51]. To this end, the antigenotoxic effect exhibited by the hydromethanolic extract of *S. verticillata* could be due to its high TPC and antioxidant activity, as well as the presence of metabolites, including rosmarinic acid. Rosmarinic acid exhibited a protective effect against the DNA damage induced by ethanol [52], and thus effectively decreased both MN formation and DNA damage in Chinese hamster lung fibroblasts (V79) treated with the chemotherapeutic agent doxorubicin [53]. Moreover, rosmarinic acid reduced the frequency of sex-linked recessive lethal mutations induced by ethyl methanesulphonate in *D. melanogaster*, and molecular docking studies showed that it physically prevents the interference of the alkylating agent with DNA [54]. It is noteworthy to mention that this study showed that the two lowest extract concentrations (10 and 25 μg mL^−1^) led to a significant reduction of the MN frequencies in MMC-treated cells, a fact that is in accordance with previous studies on phytochemicals, including the Greek plant *Pistacia lentiscus* var. *chia*, where the lowest rather than the highest extract concentrations exhibited beneficial effects [55,56].

Overall, although further studies are needed, including different cellular types, for the elucidation of the beneficial effects of plant extracts, this study revealed for the first time that the cytotoxic and antigenotoxic potential of the hydromethanolic extract of *S. verticillata* is mainly due to various phenolic acids and flavonoids, as well as their potential synergistic effects, as previously mentioned [55,57].

## 3. Materials and Methods

### 3.1. Chemicals and Reagents

All solvents (formic acid, water, acetonitrile, methanol) used for extraction and HPLC–DAD–ESI-MS and HPLC–DAD were of LC–MS and HPLC grade and were purchased from Thermo Fisher Scientific (Waltham, MA, USA). HPLC reference standards, rosmarinic acid (99%), and luteolin 7-*O*-glucoside (98.9%), were purchased from Phytolab (Vestenbergsgreuth, Germany). All the chemical reagents used for the CBMN assay are reported in our previous work [58] (see Supplementary Materials Section S3.1).

### 3.2. Plant Material

**Figure 3:**
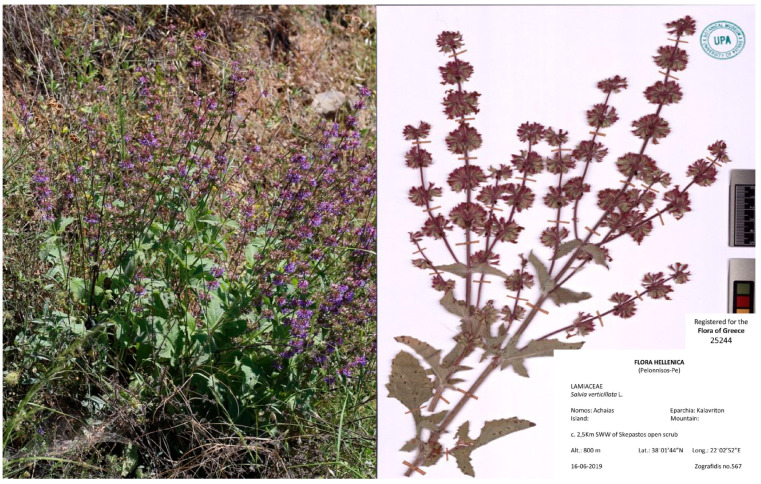
Wild plants of *Salvia verticillata* L. (**left**) and the UPA voucher specimen (**right**).

Dried leaves of *Salvia verticillata* were collected from Kalavryta, Achaia, Greece in June 2019 and were identified by the UPA Herbarium staff where the voucher was deposited (UPA 25244) (Figure 3).

### 3.3. Extraction

Two grams of dried leaves were ground and extracted with 20 mL 70% (aq.) methanol (three times) in an ultrasonic bath (120 W, 40 kHz) at a temperature below 40 °C for twenty minutes each time. After solvent removal in vacuo (rotary evaporation and freeze drying), the hydromethanolic extract was kept at –20 °C for storage. The extraction process was performed at least three times (three biological replicates).

### 3.4. High-Performance Liquid Chromatography–Diode Array Detection–Mass Spectrometry (HPLC–DAD–ESI-MS)

At the Central Laboratory of Instrumental Analysis of the University of Patras, the hydromethanolic extracts were analyzed using a Dionex UltiMate 3000 LC system (Thermo Fisher Scientific, Waltham, MA, USA) instrument coupled to a quadrupole ion-trap Bruker amaZon SL MS (Bruker Daltonics, Billerica, MA, USA) (scan time: 500 ms, fragmentor 135 V, ion scan range: 100–1000 *m*/*z* in the positive ionization and 100–2000 m/z in the negative ionization) equipped with an ESI interface (ion scan range: 100–1000 m/z in the positive ionization and 100–2000 *m*/*z* in the negative ionization). UV–vis detection was performed with a DAD detector (scan range: 190–640 nm) and was carried out at 254, 280, 330, and 380 nm. A reversed-phase C-18 Acclaim column (120 Å, 100 mm × 2.1 mm × 3 μm) from Thermo Fisher Scientific was used, and the column compartment was thermostated at 35 °C. Samples were diluted in 25% *v*/*v* methanol/water. A flow rate of 0.3 mL/min was used, and the volume of the sample injection was 10 μL. Two solvents were used for elution: A: 0.2% (*v*/*v*) formic acid in water, and B: 0.2% (*v*/*v*) formic acid in acetonitrile. The gradient elution started with 5% B for 5 min, 5–15% B during mins 5–8, with 15% B for 8 min, and then 15–22% B during mins 16–19 min, isocratic with 22% B for mins 19–30, 22–35% B for mins 30–34, 35–80% B for mins 34–50, and 95% B for mins 50–53, followed by a 9 min equilibration step at the original elution conditions. For data processing, the Bruker Compass DataAnalysis V4.2 software (Bruker Daltonics) was used. The elution order on C18 columns, UV–vis, and MS spectra were compared to the published literature on sage and rosemary samples to identify them.

### 3.5. High-Performance Liquid Chromatography–Diode Array Detector (HPLC–DAD)

The quantification was carried out on an Agilent HPLC instrument (Series 1260, Agilent Technologies Inc., Santa Clara, CA, USA) with a diode array detector (scan range: 190–640 nm) on a Kinetex 2.6 μm Polar C18 (100 Å, 100 × 3.0 mm, Phenomenex, Torrance, CA, USA) column. The UV–vis detection wavelengths were 254, 280, 330, and 380 nm, the sample concentrations were 1.0 and 0.5 mg/mL in 25% *v*/*v* methanol, the injection volume was 10 μL, and the elution flow rate was 0.4 mL/min. The rest of the procedure was the same as the HPLC–DAD–ESI-MS protocol above. Agilent Open Lab software (https://www.agilent.com/en/product/software-informatics/analytical-software-suite/chromatography-data-systems/openlab-cds accessed on 10 February 2024) was used for data processing.

One commercially available reference standard was chosen for each compound category, due to the lack of commercially available standards for all the ingredients [22,23,24]. Luteolin 7-*O*-glucoside (at 330 nm) and rosmarinic acid (at 280 nm) were used for the relative quantification of flavonols and phenolic acids, respectively. The chromatogram of the standards along with their elution times and UV–vis spectra are presented in Figure S3. The standard curves that came up were y = 22.783x + 7.7307 (R^2^ = 0.9917) for rosmarinic acid and y = 38.87x − 117.11 (R^2^ = 0.9955) for luteolin-7-*O*-glucoside. The chosen concentrations for rosmarinic acid and luteolin-7-*O*-glucoside were 5.0, 10.0, 15.6, 31.3, 46.9, 62.5, 93.8, and 150.0 µg/mL, and 4.0, 8.0, 12.5, 25.0, and 37.5 µg/mL, respectively. The lower limit of detection (LLOD) and the lower limit of quantification (LLOQ) values for the quantification of rosmarinic acid and luteolin-7-*O*-glucoside were determined using two analytical parameters: σ (standard deviation) and φ (slope of a regression curve) according to LOD = 3.3 *× σ*/*ϕ* and LOQ = 10 *× σ*/*ϕ.* The LLOD and LLOQ values were 16.20 and 49.09 μg/mL, respectively, for rosmarinic acid, and 3.507 and 10.630 μg/mL for luteolin 7-*O*-glucoside. Results are expressed as mg per g of dry extract weight and g of dry leaf.

### 3.6. Determination of Total Phenolic Content and Antioxidant Activity

To determine the total phenolic content (TPC) and antioxidant activity (AA), samples were diluted directly into the solvent used in each method. All samples were analyzed in triplicate using a TECAN Sunrise microplate reader (Tecan Group Ltd., Männedorf, Switzerland).

#### 3.6.1. Determination of Total Phenolic Content (TPC)

The TPC of the extracts was determined using the Folin–Ciocalteu method, according to the procedure described by Singleton et al. [59]. Specifically, 20 μL of extract or standard solution in H_2_O were mixed with 180 μL H_2_O, 20 μL Folin–Ciocalteu reagent and 20 μL Νa_2_CO_3_ (13.75%). Absorbance was measured at 750 nm after 30 min of incubation. Results are expressed as mg of gallic acid equivalents (GAE) per g of dry leaf (mg GAE g^−1^ dry leaf), with a standard curve (y = 0.0029x + 0.0179, R^2^ = 0.9877) of gallic acid at different concentrations (40, 60, 80, 100, 150, 200 μg/mL in H_2_O).

#### 3.6.2. Ferric Reducing Antioxidant Power (FRAP) Assay

The FRAP assay, according to Benzie and Strain [60], evaluates the power of compounds to reduce the complexes of ferric ions with 2,4,6-tripyridyl-S-triazine (TPTZ) [Fe(TPTZ)_2_]^3+^ to the ferrous ones [Fe(TPTZ)_2_]^2+^. In a 96-well microplate, a volume of 60 μL of extract or standard solution (FeSO_4_·7H_2_O) in H_2_O was put in each well, and then 55 μL of acetate buffer, pH 3.6, and 80 μL of FRAP reagent were added. The FRAP reagent was freshly prepared with a 300 mM acetate buffer, pH 3.6, 10 mM TPTZ in 40 mM HCl, and 20 mM FeCl_3_·6H_2_O in water in the following ratios: 7.5:1.5:1.5. After 5 min, the absorbance at 595 nm was measured. The results were expressed as mg of standard equivalent per g of dry leaf (mg FeSO_4_·7H_2_O g^−1^ dry weight), based on the calibration curve of FeSO_4_ (y = 4.2498x − 0.2262, R^2^ = 0.9988).

#### 3.6.3. Radical Scavenging Activity by DPPH• Assay

A modified method by Brand-Williams et al. [61] for DPPH radical scavenging was used to determine the radical scavenging activity of the samples. In a 96-well microplate, 100 μL of the sample was placed in 70% *v*/*v* methanol/water or standard solution. The DPPH reagent (100 μL, 0.3 mM in methanol) was added. After 30 min, the absorbance at 540 nm was recorded [62,63]. Butylhydroxytoluene (>99%, Sigma-Aldrich, St. Louis, MA, USA) was used as a standard, and was dissolved in methanol at concentrations of 10, 20, 30, 50, 100, 150, 175, and 225 μg/mL. The percentage of the radical scavenging activity (RSA) was calculated by the equation:

% RSA = [1 − (A_sample_ − A_blank_)/(A_control_ − A_blank_)] × 100

The IC_50_ (*n* = 3), i.e., the concentration (μg/mL) of the extract that induces a 50% reduction of DPPH radical concentration, was calculated using a sigmoidal dose-response curve, calculated using GraphPad Prism 6 (GraphPad Software, La Jolla, CA, USA).

### 3.7. CBMN Assay in Human Lymphocytes In Vitro

#### 3.7.1. Ethics Statement

The research received approval from the Research Ethics Committee (REC) of the University of Patras (UPAT) (Ref. No. 7682/23.03.2022). Blood samples were acquired from two healthy non-smoking male donors (<30 years old), who were not exposed to radiation, drug treatment or any viral infection in the recent past.

#### 3.7.2. CBMN Assay Application

The in vitro cytokinesis block micronucleus (CBMN) assay with cytochalasin-B was applied to assess the cytotoxic and genotoxic potential of the hydromethanolic *Salvia verticillata* extract in human lymphocytes (see Supplementary Materials Section S3.7.2), according to standard procedures. Moreover, the antigenotoxic potential of the extract was investigated in the presence of MMC, a well-known and widely used mutagenic agent [19]. The cytokinesis block proliferation index (CBPI) was calculated for the assessment of cytotoxicity by counting 1000 cells for each experimental point, using the equation:

CBPI = [N1 + N2 + 3(N3 + N4)]/N,

where N1, N2, N3, and N4 represent the numbers of cells with one, two, three, and four nuclei, while N is the total number of cells [64].

#### 3.7.3. Statistical Analysis

The statistical analysis was conducted according to previous work [58] (see Supplementary Materials Section S3.7.3).

## 4. Conclusions

The phytochemical profile, the total phenolic content, and the antioxidant activity of the hydromethanolic extract of *Salvia verticillata* leaves were evaluated. The results indicate that the hydromethanolic extract of *S. verticillata* leaves possesses a high content of phenolics and significant antioxidant activity. It is rich in rosmarinic acid, but there are several unknown ingredients, and this finding justifies our choice to investigate the safety of the leaf extract, since it has beneficial biological properties and the potential for numerous applications. Regarding the assessment of its cyto-genotoxic and antigenotoxic potential, this study showed an absence of genotoxic potential, despite the slight cytotoxicity in lymphocytes tested, in addition to antigenotoxic activity mainly due to the extract’s high phenolic and flavonoid content. *S. verticillata* has not been extensively studied for its beneficial properties compared to other *Salvia* species such as *Salvia officinalis*. Furthermore, most studies investigating the phytochemical and pharmaceutical properties of *S. verticillata* have used either ethanolic (or hydroethanolic) or methanolic extracts, whereas we assessed the profile of a hydromethanolic extract which could exert different activities and have alternate phytochemical characteristics. Thus, the differences in the number and type of compounds found via our characterization methods highlight the importance of both the extraction solvent(s) used and the collection area (different countries, geological and environmental conditions). Moreover, its antioxidant potential and phenolic content was found to be higher than other *Salvia* species, as well as than other *S. verticillata* polar extracts, which could prove its superiority in medicinal and pharmaceutical applications. Furthermore, this is the first study investigating the genotoxic potential of hydromethanolic *S. verticillata* extract. Apart from that, the importance of dosage in natural and/or plant extracts is emphasized, since smaller doses seem to exert a higher antigenotoxic effect compared to the highest concentration. Consequently, the beneficial properties of the studied extract and its constituents should be further explored, potentially rendering it valuable in medicinal applications and pharmaceutical products.

## Figures and Tables

**Table 1:** Tentative identification of polar secondary metabolites in hydromethanolic extracts of *Salvia verticillata* leaves using HPLC–DAD–ESI-MS. The retention times (RT), the molecular weight (MW), the ions after positive and negative ionization, and the UV–vis absorption maxima (**λ_max_**) are presented in the columns, along with the references to the previous studies that aided in the characterization.

	RT (min)	Tentative Identification	MW	Ions (*m*/*z*) after Negative Ionization	Ions (*m*/*z*) after Positive Ionization	λ_max_ (nm)	Ref.
1	1.8	quinic, citric, or isocitric acid	192	111 191 [M−H]^−^	-	200, 209sh, 215	[25]
2	4.3	dimer-β-(3,4-dihydroxyphenyl) lactic acid	396	135 [caffeic acid−H−CO_2_]^−^ 179 [caffeic acid−H]^−^ 197 [M−2H]^2−^ 395 [M−H]^−^ 417 [M+Na−2H]^−^	221 [M+2Na]^2+^ 317 435 [M+K]^+^ 831 [2M+K]^+^	198, 225, 282sh	[26]
3	8.0	caftaric acid	312	149 [tartaric acid−H]^−^ 179 [caffeic acid−H]^−^ 311 [M−H]^−^ 333 [M+Na−2H]^−^	335 [M+Na]^+^ 354 [M+ACN+H]^+^ 376 [M+ACN+Na]^+^	202, 215, 325sh	[26]
6	10.5	coumaroyl-hexose	326	163 [M-hexose]^−^ 193 325 [M−H]^−^ 371 [M+FA−H]^−^	349 [Μ+Νa]^+^ 365 [M+K]^+^ 675 [2M+Na]^+^	202sh, 219, 285, 328	[7,8,23]
7	10.7	medioresinol	388	177 256 387 [M−H]^−^ 423 [M+Cl]^−^ 433 [M+FA−H]^−^ 775 [2M−H]^−^	227 389 [M+H]^+^ 411 [Μ+Νa]^+^ 427 [M+K]^+^ 799 [2M+Na]^+^	226sh, 315	[22,23]
10	19.3	luteolin glucuronide	462	421 461 [M−H]^−^ 483 [M+Na−2H]^−^ 923 [2M−H]^−^	287 [M-glucuronide+H]^+^ 463 [Μ+H]^+^ 485 [M+Na]^+^ 925 [2M+H]^+^ 947 [2M+Na]^+^	200sh, 219, 268sh, 344	[7,8,22,24,28]
11	21.2	sagerinic acid	720	539 719[M−H]^−^ 741 [M+Na-2H]^−^	163 313 328 380 [M+H+K]^2+^ 523 721 [M+H]^+^ 743 [Μ+Νa]^+^ 759 [M+K]^+^ 1441 [2M+H]^+^ 1463 [2M+Na]^+^	199, 221, 283sh	[8,24]
12	21.9	apigenin glucuronide	446	269 [M−glucuronide−H]^−^ 445 [M−H]^−^ 891 [2M−H]^−^	271 [M−glucuronide+H]^+^ 447 [Μ+H]^+^ 469 [M+Na]^+^ 893 [2M+H]^+^	200sh, 221, 268, 337	[7,8,23,28,29]
13	22.1	rosmarinic acid	360	161 179 [M−2H]^2−^ and [caffeic acid−H]^−^ 197 359 [M−H]^−^ 719 [2M−H]^−^ 1079 [3M−H]^−^	163 361 [M+H]^+^ 383 [Μ+Νa]^+^ 721 [2M+H]^+^ 743 [2M+Na]^+^	199sh, 236, 327, 333	[7,8,25,26,28,30,31,32]
14	22.8	hispidulin glucuronide	476	299 [M−glucuronide-H]^−^ 475 [M−H]^−^ 535 [M+Hac−H]^−^ 951 [2M−H]^−^	301 [M-glucuronide+H]^+^ 477 [Μ+H]^+^ 499 [M+Na]^+^ 953 [2M+H]^+^	200, 223, 343sh	[23,24]
16	23.9	salvianolic acid K or isomer	556	493 535 555 [M−H]^−^ 577 [M+Na-2H]^−^	267 298 [M+H+K]^2+^ 323 341 359 539 557 [M+H]^+^ 559 579 [M+Na]^+^ 595 [M+K]^+^ 1155 [2M+ACN+H]^+^	225, 286sh, 325sh	[8,23]
17	24.2	salvianolic acid H,I,J, lithospermic acid, or 3′-*O*-(8″-Z-caffeoyl)rosmarinic acid	538	537 [M−H]^−^ 559 [M+Na−2H]^−^ 1075 [2M−H]^−^	457 561 [M+Na]^+^ 1077 [2M+H]^+^ 1099 [2M+Na]^+^ 1115 [2M+K]^+^	199, 223, 286sh, 320sh	[24,28,32]
18	26.0	salvianolic acid E	718	537 717 [M−H]^−^ 739 [M+Na−2H]^−^	323 379 [M+H+K]^2+^ 521 719 [M+H]^+^ 741 [Μ+Na]^+^ 757 [M+K]^+^ 1459 [2M+Na]^+^ 1475 [2M+K]^+^	199, 223, 286sh, 320sh	[25,26,32,33]
19	26.5	salvianic acid A (danshensu) rhamnoside	344	179 [danshensu]^−^ 243 343 [M−H]^−^ 379 [M+Cl]^−^ 687 [2M−H]^−^ 733 [2M+FA−H]^−^ 747 [2M+Hac−H]^−^	147 367 [Μ+Na]^+^	226, 286sh, 320sh	[25,32]
20	27.9	*cis*-methyl rosmarinic acid	374	135 179 373 [M−H]^−^ 395 [M+Na−2H]^−^ 747 [2M−H]^−^	177 341 397 [Μ+Na]^+^ 771 [2M+Na]^+^	197, 223, 286sh, 326sh	[8,25,26,34]
21	28.3	*trans*-methyl rosmarinic acid	374	373 [M−H]^−^ 747 [2M−H]^−^ 1120 [3M−H]^−^	177 375 [M+H]^+^ 397 [Μ+Na]^+^ 771 [2M+Na]^+^ 787 [2M+K]^+^	199sh,220,285sh, 329	[7,8,25,26]
22	33.8	salvianolic acid B, L or isosalvianolic acid B	718	717 [M−H]^−^ 739 [M+Na−2H]^−^	323 343 371 [M+H+Na]^2+^ 379 [M+H+K]^2+^ 719 [M+H]^+^ 741 [Μ+Na]^+^ 1454 [2M+NH_4_]^+^	198, 224, 284sh, 330sh	[23,32,33]
23	34.7	dedihydro- salvianolic acid B/isomer	716	715 [M−H]^−^ 737 [M+Na−2H]^−^	295 378 [M+H+K]^2+^ 519 [salvianolic acid B-H-98]^−^ 739 [Μ+Na]^+^ 755 [M+K]^+^ 1455 [2M+Na]^+^	199sh, 227, 284sh, 346	[33]
25	38.8	circimaritin	314	283 [M−H−2CH_3_]^−^ 313 [M−H]^−^	315 [Μ+H]^+^ 337 [M+Na]^+^ 651 [2M+Na]^+^	227, 277sh, 335	[7,31]
26	43.4	salvigenin	328	-	329 [M+H]^+^ 351 [M+Na]^+^ 392 [M+ACN+Na]^+^ 679 [2M+Na]^+^	228, 280sh, 332	[29,31]
28	48.6	miltipolone or hinokione	300		149 205 301 [M+H]^+^	228, 278sh	[31,35]

Peak numbering in this Table and in Table S1 is uniform according to the elution order. The unknown compounds are presented in Table S1. Abbreviations: ACN, acetonitrile; FA, formic acid; Hac, acetic acid.

**Table 2:** Concentrations of the major polar metabolites in *Salvia verticillata* extract. The first column shows the peak number of the analyte as presented in Table 1. Three biological samples were analyzed twice (*n* = 6).

	**Wavelength (nm)**	**Compound**	**Concentration** **(mg g^−1^ Dry Extract Weight)**	**Concentration** **(mg g^−1^ Dry Leaf)**
**Phenolic acids ^a^**				
8	280	unknown (MW = 592)	13.44 ± 1.63	5.16 ± 0.63
13	280	rosmarinic acid	223.12 ± 8.66	85.63 ± 3.32
21	280	*trans*- methyl rosmarinic acid	63.03± 7.33	24.19 ± 2.81
23	280	dedihydro-salvianolic acid B/isomer	19.42 ±2.81	7.45 ± 1.08
**Flavonoids ^b^**				
10	330	luteolin-glucuronide	23.92 ± 2.20	9.18 ± 0.84
12	330	apigenin-glucuronide	29.71 ± 4.33	11.40 ± 1.66
14	330	hispidulin glucuronide	6.97 ± 0.67	2.68 ± 0.26
25	330	circimaritin	6.85 ± 0.80	2.63 ± 0.31
26	330	salvigenin	5.27 ± 0.47	2.02 ± 0.18

Phenolic acids were quantified in relation to rosmarinic acid (superscript letter a), and flavonoids to luteolin 7-*O*-glucoside (superscript letter b).

## Data Availability

Data are contained within the article.

## Supplementary Materials

The following supporting information can be downloaded at: https://www.mdpi.com/article/10.3390/plants13050731/s1, Section S3.1: Chemicals and reagents, Section S3.7.2: CBMN assay application, Section S3.7.3: Statistical analysis, Table S1: Unknown compounds in hydromethanolic extracts of *Salvia verticillata* leaves using HPLC–DAD–ESI-MS. The retention times (RT), the molecular weight (MW), the ions after positive and negative ionization, and the UV–vis absorption maxima are presented in the columns. Peak numbering herein and in Table 1 is according to the elution time. Figure S1: Chromatogram at 280 nm with HPLC–DAD–ESI-MS of an extract of *S. verticillata* leaves (3 mg/mL). Peak numbering is as in Table 1 and Table S1. Figure S2: Chromatograms (HPLC–DAD) at 280 nm (upper panel) and at 330 nm (bottom panel) of an extract of *S. verticillata* leaves (3 mg/mL). Peak numbering is as in Table 2, Figure S3: Chromatogram (HPLC–DAD) at 330 nm of the two standard compounds: luteolin-7-*O*-glucoside (12.5 μg/mL) and rosmarinic acid (31.3 μg/mL).

## Abbreviations

TPC	Total Phenolic Content
AA	Antioxidant Activity
FRAP	Ferric Reducing Antioxidant Power
RT	Retention Times
MW	Molecular Weight
CBMN	Cytokinesis Block MicroNucleus
MMC	Mitomycin C
MN	Micronuclei
CBPI	Cytokinesis Block Proliferation Index
MTT	3-(4,5-dimethylthiazol-2-yl)-2,5-diphenyl tetrazolium bromide
