# Supplementary material for: Phytochemical Profile and Evaluation of the Antioxidant, Cyto-Genotoxic, and Antigenotoxic Potential of Salvia verticillata Hydromethanolic Extract
Source: Plants (Basel). 2024 Mar 5;13(5):731. doi: 10.3390/plants13050731 (PMC10934106; doi:10.3390/plants13050731)
Supplement: Supplementary file 1 [file plants-13-00731-s001.zip › plants-2892122-supplementary.pdf]

Supplementary Materials

Phytochemical profile and evaluation of the antioxidant, cyto-genotoxic and antigenotoxic potential of *Salvia verticillata* hydromethanolic extract

Lamprini S. Stavropoulou[1], Ioanna Efthimiou[2], Lambrini Giova[2], Chrysoula Manoli[2], Paraskevi S. Sinou[1], Aris Zografidis[3], Fotini N. Lamari[1], Dimitris Vlastos[2], Stefanos Dailianis[2], Maria Antonopoulou[4*]

[1]*Laboratory of Pharmacognosy & Chemistry of Natural Products, Department of Pharmacy, University of Patras, GR-26504 Patras, Greece*

[2]*Department of Biology, School of Natural Sciences, University of Patras, GR-26504 Patras, Greece*

[3]*Laboratory of Botany, Department of Biology, University of Patras, GR-26504 Greece*

[4]*Department of Sustainable Agriculture, University of Patras, GR- 30131 Agrinio, Greece*

*Corresponding author

Email address: mantonop@upatras.gr

SM. Materials and Methods

SM 3.1 Chemicals and reagents

HAM's F-10 medium, L-glutamine, Foetal bovine serum (FBS) and Phytohaemaglutinin (PHA) were supplied from Gibco, UK. Mitomycin C (MMC) and Giemsa were purchased from Sigma-Aldrich Chemical Co. (St. Louis, MO, USA) and Cytochalasin-B (Cyt-B) from Santa-Cruz. All other solvents and chemicals used were of the highest grade commercially available. The stocks of the compounds and solutions were kept at 4 °C until use.

SM 3.7.2 CBMN assay application

CBMN assay was performed according to OECD 487 Guideline (2016). Specifically, 0.5 mL of whole blood from each donor were added in 6.5 mL Ham's F-10 medium, including 1.5 mL foetal bovine serum (FBS) and 0.3 mL phytohaemagglutinin (PHA), in order to stimulate cell division. All samples were placed at 37 °C, under 5 % CO_2, for 24 h (Thermo Scientific Incubator). 24 h later, *S. verticillata* extract was added in final concentrations of 10, 25 and 50 μg mL^{-1} with and without the presence of MMC (0.05 μg mL^{-1}). After 20 h of culture treatment with the aforementioned concentrations, cytochalasin-B (Cyt-B, at a final concentration of 6 μg mL^{-1}) was added, for the prevention of cytokinesis (inhibition of actin polymerization), thus favoring nuclear division and the formation of binucleated (BN) cells with low baseline micronuclei frequency. All cultures were incubated for 72 h in total. Then, cells were harvested, collected by centrifugation (252×g, 10min) and treated for 3 min with a mild hypotonic solution (Ham's medium and Milli-Q water, ratio 3:1) at room temperature, followed by fixation with freshly prepared methanol/acetic acid (ratio 5:1, in triplicate). Finally, cells were stained with Giemsa 10 % v/v and prepared for further analysis. CBPI was evaluated by counting at least 1000 cells in any case, following the equation:

$$CBPI = \frac{(N1 + 2 \times N2 + 3 \times N3, N4)}{N}$$

where N1, N2, N3 and N4 correspond to the numbers of cells with one, two, three and four nuclei and N is the total number of cells (Surrallés et al., 1995). MN frequency in BN cells was evaluated automatically, using the MNScore slide-scanning platform of the Metafer system (MetaSystems, Altlussheim, Germany). The latter includes a motorized microscope (AxioImager Z1, Carl Zeiss, Jena, Germany) with fluorescence illumination, a motorized X/Y scanning stage (Maerzhaeuser, Wetzlar, Germany) with a range of 225 × 76mm, a high-resolution monochrome megapixel charge-coupled device

(CCD) camera (M4+; JAI AS, Glostrup/Copenhagen, Denmark), and a Windows™ compatible PC (DELL, Langen, Germany) with the Metafer software. At least 2000 BN cells with preserved cytoplasm were scored in any case, following well-established criteria (OECD, 2016).

SM 3.7.3 Statistical analysis

The final data are expressed as mean ± standard deviation (SD) of two independent experiments. Statistical analysis was conducted using the software package SPSS 25 (IBM Inc., Armonk, NY, USA, 20190). Data sets were checked for the assumptions of normality (Shapiro-Wilk W Test) and of variance homogeneity (Levene's test). Statistically significant differences between variables obtained in control and treated cells were evaluated non-parametrically, using the Mann-Whitney U-test ($p < 0.05$).

SM. Results

Table S1. Unknown compounds in hydromethanolic extracts of *Salvia verticillata* leaves using HPLC–DAD–ESI–MS. The retention times (RT), the molecular weight (MW), the ions after positive and negative ionization, and the UV–vis absorption maxima are presented in the columns. Peak numbering herein and in Table 1 is according to the elution time.

	RT (min)	Tentative identification	MW	Ions (m/z) after negative ionization	Ions (m/z) after positive ionization	λ_{max} (nm)
4	9.4	unknown	282	281 $[M{-}H]^-$<br>563 $[2M{-}H]^-$	164 $[M+2Na]^{2+}$<br>305 $[M+Na]^+$	203sh, 219, 329
5	10.2	unknown	-	135, 179, 265, 325, 387, 459, 479, 523, 538, 560	349, 540	202sh, 220, 323
8	11.7	unknown	592	207<br>295 $[M{-}2H]^{2-}$<br>319<br>591 $[M{-}H]^-$	177<br>199<br>297 $[M+2H]^{2+}$<br>319 $[M+2Na]^{2+}$<br>615 $[M+Na]^+$<br>1223 $[2M+K]^+$	198sh, 219, 329
9	13.4	unknown	556	537 $[M{-}H_2O{-}H]^-$<br>555 $[M{-}H]^-$<br>577 $[M+Na{-}2H]^-$	182<br>298 $[M+H+K]^{2+}$<br>323<br>341<br>539<br>579 $[M+Na]^+$<br>1135 $[2M+Na]^+$	198, 224, 277sh
15	23.4	unknown(s)	734	461<br>513<br>537<br>548<br>581<br>703<br>719<br>733 $[M{-}H]^-$	191<br>277<br>295<br>387 $[M+H+K]^{2+}$<br>515<br>537<br>558<br>735 $[M+H]^+$	198, 223, 285sh, 320sh

				755[M+Na−2H]$^-$	757 [M+Na]$^+$	
24	36.1	unknown	730	343<br>405<br>717<br>729 [M−H]$^-$<br>751 [M+Na−2H]$^-$	296<br>312<br>328<br>345<br>377[M+H+Na]$^{2+}$<br>385[M+H+K]$^{2+}$<br>429<br>533<br>731[M+H]$^+$<br>748[M+NH$_4$]$^+$<br>753[M+Na]$^+$<br>769[M+K]$^+$<br>1483[2M+Na]$^+$	198, 226, 290sh,<br>338sh
27	46.8	unknown	296	277<br>295 [M−H]$^-$	149 [M+2H]$^{2+}$<br>243<br>279<br>297 [M+H]$^+$<br>319 [M+Na]$^+$	228, 278sh

Abbreviations: ACN, acetonitrile; FA: formic acid; Hac: acetic acid

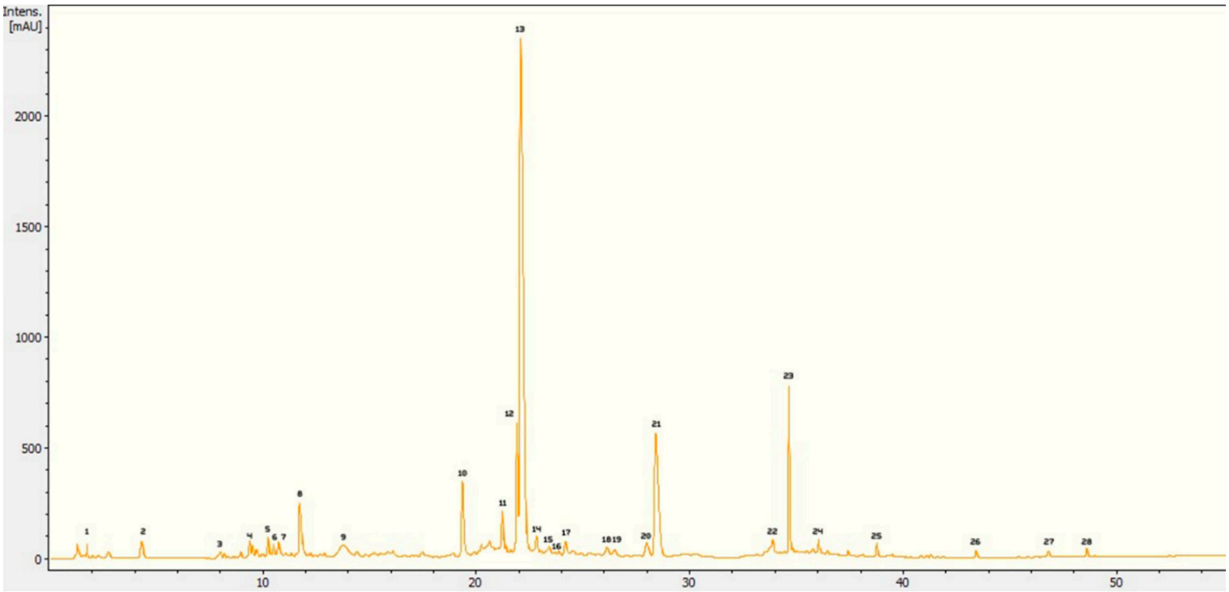

Figure S1. Chromatogram at 280 nm with HPLC-DAD-ESI-MS of an extract of *S. verticillata* leaves (3 mg/mL). Peak numbering as in Table 1 and Table S1.

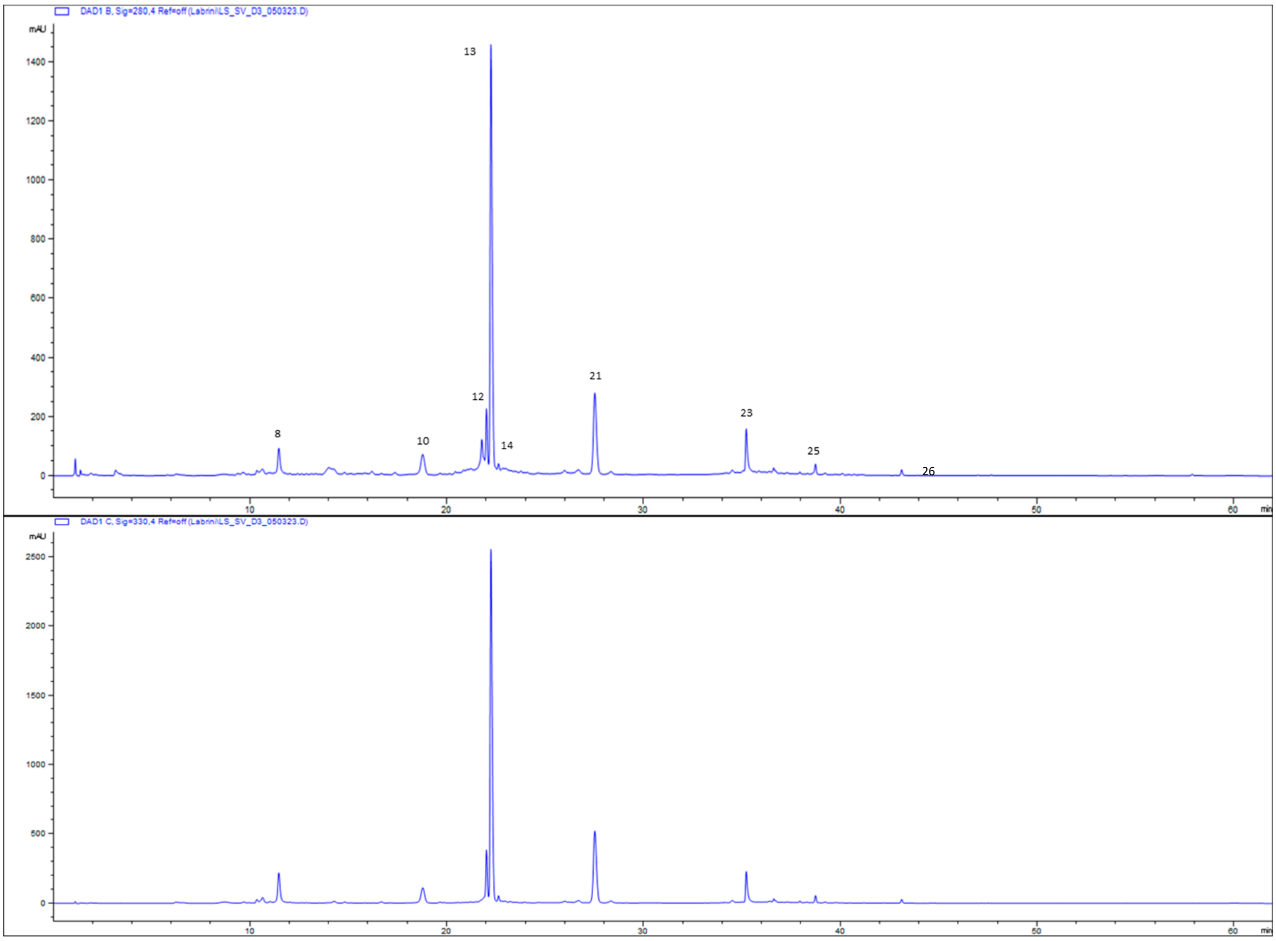

Figure S2. Chromatograms (HPLC-DAD) at 280 nm (upper panel) and at 330 nm (bottom panel) of an extract of *S. verticillata* leaves (3 mg/mL). Peak numbering as in Table 2.

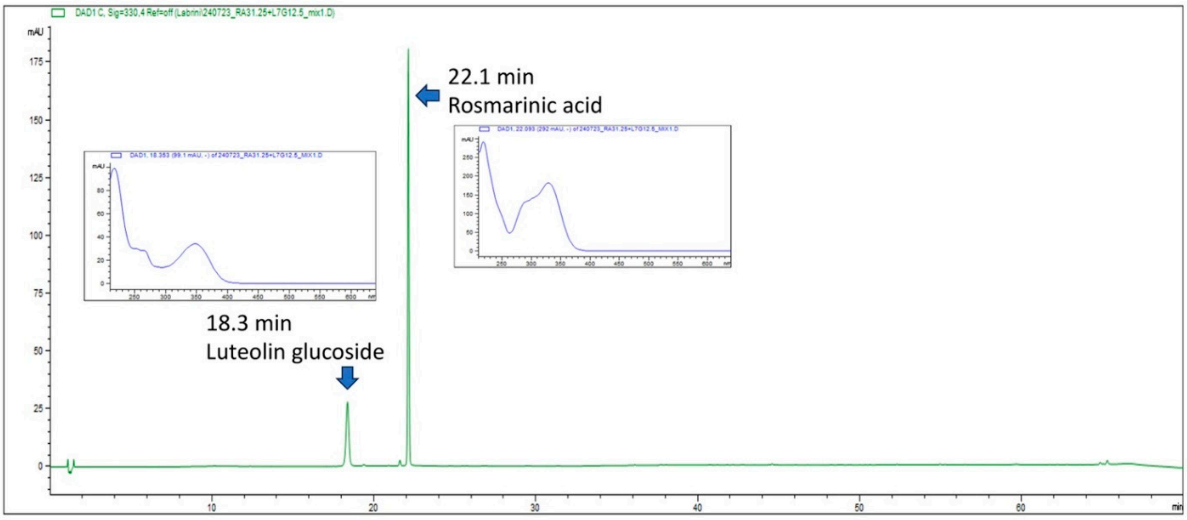

Figure S3. Chromatogram (HPLC-DAD) at 330 nm of the two standard compounds: luteolin-7-*O*-glucoside (12.5 µg/mL) and rosmarinic acid (31.3 µg/mL).

SM. References

OECD. Test No. 487: In Vitro Mammalian Cell Micronucleus Test. In OECD Guidelines for the Testing of Chemicals; Section 4; OECD Publishing: Paris, France, 2016.

Surrallés, J.; Xamena, N.; Creus, A.; Catalán, J.; Norppa, H.; Marcos, R. Induction of Micronuclei by Five Pyrethroid Insecticides in Whole-Blood and Isolated Human Lymphocyte Cultures. *Mutat. Res.* **1995**, *341*, 169–184, doi:10.1016/0165-1218(95)90007-1.
